# Tourism Revenue as a Conservation Tool for Threatened Birds in Protected Areas

**DOI:** 10.1371/journal.pone.0062598

**Published:** 2013-05-08

**Authors:** Rochelle Steven, J. Guy Castley, Ralf Buckley

**Affiliations:** International Centre for Ecotourism Research, Griffith University, Gold Coast, Queensland, Australia; University of Western Ontario, Canada

## Abstract

Many bird populations worldwide are at risk of extinction, and rely heavily on protected area networks for their continued conservation. Tourism to these areas contributes to conservation by generating revenue for management. Here we quantify the contribution of tourism revenue for bird species in the IUCN Red List, using a simple accounting method. Relevant data are available for 90 (16%) of the 562 critically endangered and endangered species. Contributions of tourism to bird conservation are highest, 10–64%, in South America, Africa, and their neighbouring islands. Critically endangered bird species rely on tourism more heavily than endangered species (*p*<0.02). Many protected areas could also enhance their management budgets by promoting birdwatching tourism specifically.

## Introduction

Approximately 13% of extant bird species are threatened with extinction [1], [2] and protected area (PA) networks are the mainstay of global efforts to conserve them [2], [3], [4]. These networks include: formal gazetted public national parks and reserves (e.g. IUCN Category II); internationally designated areas such as Biosphere Reserves; communal conservancies; and private reserves [5], [6]. There are >120,000 public protected areas worldwide, covering ∼13% of global terrestrial habitats [4]. Some bird species remain extant only as a result of reserves designated and managed specifically to conserve them [7], [8].

Despite previous efforts, current protected area networks provide incomplete protection for global biodiversity, and species continue to decline in diversity and abundance [3], [9]. Reversing these trends of decline requires a thorough understanding of species ecology, threatening processes, as well as the expansion and active management of global protected area networks [3], [9], [10], [11]. These approaches require funds for conservation measures such as habitat restoration, control of invasive species, reintroductions of threatened species, and anti-poaching efforts [3], [9], [10], [12], [13]. Generally, funds for conserving biodiversity in protected areas worldwide remain inadequate to meet these needs [9], [14].

Many protected area managers seeking to expand and diversify their funding portfolios consider tourism revenue to be an increasingly significant fiscal source for protected area management and conservation. Revenue is raised from entrance and activity charges, accommodation, concession and lease fees, and sales of tourist commodities [6], [13], [15], [16]–[22]. Additional indirect contributions to conservation may occur through various social mechanisms. These include educating tourists and changing their behaviours; providing benefits to local communities to reduce dependence on natural resources [23]–[26]; and improving local awareness and attitudes towards conservation [12], [24], [25].

However, tourism can also create negative impacts in protected areas, affecting both the environment and species within them [16], [27], [28]. The scale, extent and severity of these impacts depend on the nature of the tourism activity. Previous studies have highlighted the variable impacts to birds from both motorised and non-motorised tourism activities [27], [28]. So long as these impacts are managed effectively, tourism can deliver net benefits to species and ecosystems [6], [15], [20], [29], [30] but these are rarely quantified.

The relationship between tourism revenue to protected areas, and conservation of the threatened species which occur in them, has been demonstrated recently for mammals and frogs at the global scale [29], [30], but not for any other taxa. Here, therefore, we examine firstly, the contributions of protected areas networks to conservation of threatened bird species; and secondly, the degree to which tourism revenue to protected areas contributes to the conservation of critically endangered and endangered bird species. We hypothesise that for threatened birds in developing countries, where government funding for conservation is scarce, tourism revenue plays a key role in conservation funding.

## Methods

The degree to which individual threatened species depend on tourism revenues to protected areas can be quantified using simple accounting approaches, either for species populations [29] or species ranges [30]. Here we apply the population accounting approach [29], to all 562 bird species classified as critically endangered (CR) or endangered (EN) in the IUCN Red List of Threatened Species [31]. We use the widely adopted IUCN codes, CR and EN respectively, to refer to these threat classifications when reporting results [1], [4], [29], [30]. For each species, this approach calculates *T*, the proportion of global population protected by tourism, as *T = (Σ_1_^n^S_n_R_n_)/G* where *S_n_* are the sizes of subpopulations in parks, *R_n_* are proportions of parks revenue from tourism, and *G* is the global population for the species concerned [29].

We compiled data on the population status and distributions for each of these 562 threatened bird species from the IUCN Red List and BirdLife International databases [32]. As in previous studies [1], [4], [10], [29], [30] we used these data sources and classifications as they provide a consistent and reliable measure of threat status at the global scale, despite their inherent limitations. In calculating *S_n_*, we included only resident subpopulations [31], [32] within each protected area. For migratory species, we included only those protected areas which are significant breeding sites. We included all protected areas which support known populations of critically endangered or endangered bird species, including those where tourism revenue, *R* = 0. We obtained data on *R* from parks agency financial statements, annual reports and published compendia [21], [22], [29], [30] for ∼35 of the world's 196 sovereign nations (Table S1). Such financial data for individual protected areas across global scales are rarely available or consistent. Therefore, we followed approaches used for mammals and frogs [29], [30] and calculated *R* at national scale, since protected area agencies routinely transfer funds between individual parks.

## Results

There are 190 CR and 372 EN bird species in the IUCN Red List of Threatened Species, 562 species in total. For 109 CR and 132 EN species, 57% and 35% respectively, there are no records of occurrence in any protected area worldwide. The distributions of the remaining 81 CR and 240 EN species intersect 520 protected areas in 77 countries. Of these, 17 CR and 11 EN species survive in only a single protected area. Four species survive only in Galapagos National Park in Ecuador; three on Norfolk Island in Australia; two in Junin National Reserve in Peru; and two in Alakai Wilderness Preserve in Hawaii, USA (Table S2). All remaining CR and EN bird species, 34% and 62% respectively, currently survive in at least two individual protected areas.

Worldwide, 413 protected areas conserve only a single CR or EN bird species, and 107 protected areas in 33 countries support two or more species (Table S3). Galapagos National Park supports eight CR and EN species; and Pedra Talhada Biological Reserve in Brazil, and Gough Island Nature Reserve in the southern Atlantic Ocean each support six (Table 1). This highlights the relatively high numbers of threatened bird species supported in island protected areas worldwide.

**Table 1:** Protected areas where four or more critically endangered (CR) or endangered (EN) bird species are recorded.

Reserve Name	Country	Total number of CR or EN bird species recorded in this reserve	Number of CR or EN species recorded only in this reserve
Galapagos National Park	Ecuador	8	4
Pedra Talhada Biological Reserve	Brazil	6	0
Gough Island Nature Reserve	Saint Helena	6	1
Munchique National Natural Park	Colombia	5	0
South East Island (Rangatira) Nature Reserve	New Zealand	5	1
Emas National Park	Brazil	4	0
Frei Caneca Private Reserve	Brazil	4	0
Sooretama Biological Reserve	Brazil	4	0
Sierra Nevada de Santa Marta Biosphere Reserve	Colombia	4	1
French Southern and Antarctic Lands	France	4	0
Black River Gorges National Park	Mauritius	4	0
Fiordland National Park	New Zealand	4	0
Mangere Island Nature Reserve	New Zealand	4	0
Mt Canlaon Natural Park	Philippines	4	0

Tourism revenue contributions to protected area budgets, *R*, range from zero in some countries to 100% for a number of private reserves. North Island in the Seychelles, and Cerro Batipa Private Reserve in Panama, provides examples of the latter (Table S1). For most of the countries relevant to this analysis, *R* at national scale ranges from 5–80%. For Australia, New Zealand and the USA, *R*<10%, and for Canada, *R* = 14%. For the 19 South and Central American countries where data are available, *R*>20% for five, 10%<*R*<20% for five, and *R*<10% for nine. Of the six continental African countries where population data *S_n_* are known for CR and EN bird species, data on *R* are available for five, where *R* ranges from between 36–81%. Globally, *R* is higher on average for developing than developed nations.

Subpopulation data for threatened bird species in protected areas, *S_n_*, are available for 55 CR and 85 EN species, comprising 216 subpopulations in all. Data for both *S* and *R* are available for 91 species in 131 subpopulations. For four species, data on *R* are available for only some of the relevant protected areas. These species are the Seychelles Magpie-robin *Copsychus sechellarum*, Seychelles White-eye *Zosterops modestus*, Black-footed Albatross *Phoebastria nigripes*, and Sooty Albatross *Phoebetria fusca*. Calculations of *T* for these species thus reflect only some of the known subpopulations.

**Figure 1:**
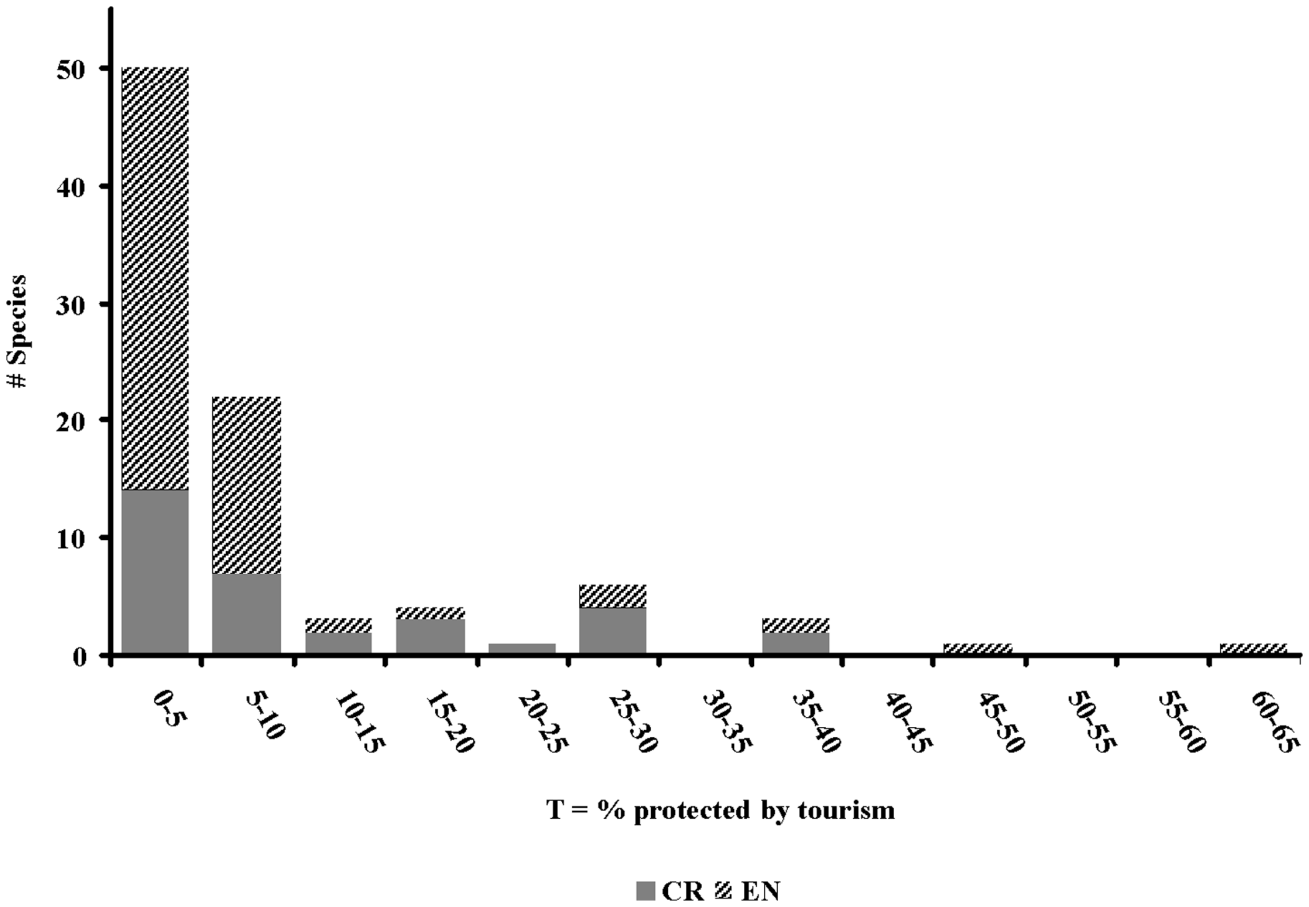
Proportions *T* of CR and EN bird species with conservation funding from tourism.

**Figure 2:**
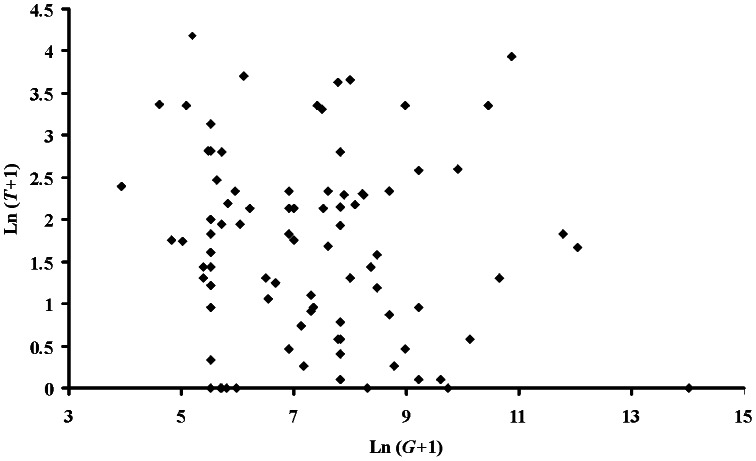
Relation between *T* and *G*, natural log scales, for CR and EN bird species (n = 91).

For these 91 species, *T* ranges from 0–64% (Table 2, Fig. 1). For nine species, *T* = 0, as the protected areas in which these species occur do not receive income from tourism. For 41 species: 0%<*T*<5%; for 21 species, 5%≤*T*<10%; for 8 species, 10%≤*T<*20%; and for 12 species, *T*>20% (Fig. 1). Mean *T* is significantly greater for CR than EN bird species (arcsine square-root transformation, *t* = 0.082, d.f. = 89, *p*<0.02). For two species, *T*≥50%: the Seychelles Magpie-robin (*T* = 64%) and the African Penguin (*T* = 50%). It is not only small populations that rely on tourism revenue since there is no correlation between *T* and global populations *G* (*R^2^* = 0.008, *d.f.* = 89, *p* = 0.397) (Fig. 2). For example, the Seychelles Magpie-robin and African Penguin with *T*≥50% have global population sizes *G* of 100–500 and 50,000–100,000 respectively.

**Table 2:** Proportions of critically endangered (CR) and endangered (EN) bird populations protected through tourism-related revenue.

Scientific Name	Common Name	IUCN	Global population (*G*)	Number* of protected subpopulations with known *S, R*	Number of individuals protected through tourism (*SR*)	Percentage of global population protected through tourism (*T*)
*Copsychus sechellarum*	Seychelles Magpie-robin	EN	178	2	114	64.0
*Spheniscus demersus*	African Penguin	EN	52000	6	26040	50.1
*Zosterops modestus*	Seychelles White-eye	EN	450	3	178	39.6
*Sephanoides fernandensis*	Juan Fernandez Firecrown	CR	3000	1	1137	37.9
*Malaconotus alius*	Uluguru Bush-shrike	CR	2400	1	881	36.7
*Mimus trifasciatus*	Floreana Mockingbird	CR	100	1	28	28.0
*Camarhynchus pauper*	Medium Tree-finch	CR	1660	1	460	27.7
*Phoebastria irrorata*	Waved Albatross	CR	34700	1	9612	27.7
*Mimus melanotis*	San Cristobal Mockingbird	EN	8000	1	2216	27.7
*Camarhynchus heliobates*	Mangrove Finch	CR	160	2	44	27.5
*Spheniscus mendiculus*	Galapagos Penguin	EN	1800	1	474	26.3
*Artisornis moreaui*	Long-billed Tailorbird	CR	250	1	55	22.0
*Buteo ridgwayi*	Ridgway's Hawk	CR	240	1	38	15.8
*Polioptila clementsi*	Iquitos Gnatcatcher	CR	250	1	39	15.6
*Laterallus tuerosi*	Junin Rail	EN	2500	1	388	15.5
*Podiceps taczanowskii*	Junin Grebe	CR	304	1	47	15.5
*Hylonympha macrocerca*	Scissor-tailed Hummingbird	EN	20000	1	2480	12.4
*Pterodroma phaeopygia*	Galapagos Petrel	CR	10000	4	1219	12.2
*Terpsiphone corvina*	Seychelles Paradise-flycatcher	CR	278	2	30	10.8
*Zosterops albogularis*	White-chested White-eye	CR	50	1	5	10.0
*Grus americana*	Whooping Crane	EN	382	1	36	9.4
*Manorina melanotis*	Black-eared Miner	EN	1000	1	94	9.4
*Papasula abbotti*	Abbott's Booby	EN	6000	1	564	9.4
*Zosterops tenuirostris*	Slender-billed White-eye	EN	2000	1	188	9.4
*Ara ambiguus*	Great Green Macaw	EN	3700	3	334	9.0
*Pardalotus quadragintus*	Forty-spotted Pardalote	EN	3800	2	339	8.9
*Cacatua haematuropygia*	Philippine Cockatoo	CR	2700	2	239	8.9
*Aceros narcondami*	Narcondam Hornbill	EN	340	1	27	7.9
*Threskiornis bernieri*	Madagascar Sacred Ibis	EN	3250	1	254	7.8
*Myiotheretes pernix*	Santa Marta Bush-tyrant	EN	2500	1	190	7.6
*Anas laysanensis*	Laysan Duck	CR	1100	2	82	7.5
*Acrocephalus familiaris*	Millerbird	CR	1000	1	74	7.4
*Myadestes palmeri*	Puaiohi	CR	500	1	37	7.4
*Oreomystis bairdi*	Akikiki	CR	1840	1	136	7.4
*Crax blumenbachii*	Red-billed Curassow	EN	250	3	16	6.4
*Aratinga brevipes*	Socorro Parakeet	EN	300	1	18	6.0
*Mimus graysoni*	Socorro Mockingbird	CR	420	1	25	6.0
*Anas wyvilliana*	Hawaiian Duck	EN	2525	1	148	5.9
*Leptoptilos dubius*	Greater Adjutant	EN	1000	2	52	5.2
*Petroica traversi*	Black Robin	EN	250	2	13	5.2
*Phoebastria nigripes*	Black-footed Albatross	EN	130000	3	6700	5.2
*Strigops habroptila*	Kakapo	CR	124	2	6	4.8
*Pterodroma axillaris*	Chatham Petrel	EN	1100	1	53	4.8
*Pterodroma magentae*	Magenta Petrel	CR	150	2	7	4.7
*Psephotus chrysopterygius*	Golden-shouldered Parrot	EN	2000	2	87	4.4
*Eudyptes sclateri*	Erect-crested Penguin	EN	170000	2	7392	4.3
*Anas nesiotis*	Campbell Islands Teal	CR	250	3	10	4.0
*Fregata andrewsi*	Christmas Frigatebird	CR	4800	1	188	3.9
*Heteroglaux blewitti*	Forest Owlet	CR	250	1	8	3.2
*Pyrrhura griseipectus*	Grey-breasted Parakeet	CR	250	1	8	3.2
*Odontophorus strophium*	Gorgeted Wood-quail	EN	4300	1	137	3.2
*Porphyrio hochstetteri*	Takahe	EN	220	2	7	3.2
*Thinornis novaeseelandiae*	Shore Plover	EN	220	1	6	2.7
*Cyanoramphus malherbi*	Malherbe's Parakeet	CR	663	3	18	2.7
*Rollandia microptera*	Titicaca Grebe	EN	3000	2	81	2.7
*Phoebetria fusca*	Sooty Albatross	EN	42000	1	1133	2.7
*Carpodectes antoniae*	Yellow-billed Cotinga	EN	794	1	20	2.5
*Mergus octosetaceus*	Brazilian Merganser	CR	250	1	6	2.4
*Penelope albipennis*	White-winged Guan	CR	250	3	6	2.4
*Megadyptes antipodes*	Yellow-eyed Penguin	EN	4800	3	110	2.3
*Geothlypis beldingi*	Belding's Yellowthroat	CR	1500	1	30	2.0
*Torreornis inexpectata*	Cuban Sparrow	EN	700	1	13	1.9
*Conothraupis mesoleuca*	Cone-billed Tanager	CR	250	1	4	1.6
*Callaeas cinereus*	Kokako	EN	1538	1	24	1.6
*Pipile jacutinga*	Black-fronted Piping-guan	EN	10000	1	156	1.6
*Ardea humbloti*	Madagascar Heron	EN	1500	1	22	1.5
*Ardeola idae*	Madagascar Pond-heron	EN	6000	1	85	1.4
*Amazona vinacea*	Vinaceous Amazon	EN	2500	3	30	1.2
*Penelope perspicax*	Cauca Guan	EN	1235	2	14	1.1
*Apteryx mantelli*	Northern Brown Kiwi	EN	25000	3	202	0.8
*Amazona rhodocorytha*	Red-browed Amazon	EN	2500	1	19	0.8
*Eleothreptus candicans*	White-winged Nightjar	EN	2400	1	18	0.8
*Pedionomus torquatus*	Plains-wanderer	EN	8000	2	50	0.6
*Phytotoma raimondii*	Peruvian Plantcutter	EN	1000	1	6	0.6
*Curaeus forbesi*	Forbes's Blackbird	EN	2500	1	12	0.5
*Haliaeetus vociferoides*	Madagascar Fish-eagle	CR	250	1	1	0.4
*Eutriorchis astur*	Madagascar Serpent-eagle	EN	250	1	1	0.4
*Cistothorus apolinari*	Apolinar's Wren	EN	1300	1	4	0.3
*Anodorhynchus hyacinthinus*	Hyacinth Macaw	EN	6500	1	18	0.3
*Brotogeris pyrrhoptera*	Grey-cheeked Parakeet	EN	15000	2	22	0.1
*Anas bernieri*	Madagascar Teal	EN	2500	1	2	0.1
*Terenura sharpei*	Yellow-rumped Antwren	EN	10000	1	8	0.1
*Ara rubrogenys*	Red-fronted Macaw	EN	4000	1	1	0.0
*Diomedea sanfordi*	Northern Royal Albatross	EN	17000	1	2	0.0
*Thalassarche melanophrys*	Black-browed Albatross	EN	1220000	2	77	0.0
*Eleoscytalopus psychopompus*	Bahia Tapaculo	CR	250	1	0	0.0
*Zosterops chloronothus*	Mauritius Olive White-eye	CR	296	1	0	0.0
*Foudia rubra*	Mauritius Fody	EN	328	1	0	0.0
*Nesoenas mayeri*	Pink Pigeon	EN	395	1	0	0.0
*Psittacula eques*	Mauritius Parakeet	EN	300	1	0	0.0
*Trichocichla rufa*	Long-legged Thicketbird	EN	250	3	0	0.0

* Other subpopulations lacking data on *S* and *R* may also exist.

## Discussion

Our findings as presented above fall into two major categories: firstly, on the degree of protection or otherwise for threatened bird species; and secondly, on the relative contributions of revenue from tourism. In the first category, we show that 57% of critically endangered and 35% of endangered bird species do not occur at all inside the current global protected area network, highlighting the global extinction risk facing many species. We also show that 14 individual protected areas each protect the last remaining populations for more than one critically endangered or endangered bird species (Table 1), and are hence of particular significance for bird conservation. Half of these protected areas are in South America, reflecting the high diversity and restricted ranges of neotropical avifauna. Three are on islands, particularly vulnerable to some threats but relatively protected from others [33], and sometimes amenable to direct conservation interventions, with a number of successful examples [34]–[36]. These findings confirm longstanding concerns over inadequate protection of bird species worldwide [9], [10].

These findings are subject to limitations of available data on populations, distributions and threats to bird species. These limitations influence both the IUCN Red List classifications of species as critically endangered or endangered, and the reliability and completeness of numerical estimates for protected subpopulations, *S*_n_. However, these limitations are unlikely to compromise our findings for several reasons. The first relates to the global scale of our assessment. A number of countries, and in some case subsidiary jurisdictions such as states and provinces, maintain their own legislation for protection of threatened species, which do not necessarily match the IUCN Red List either in the species included, the categories of threat status, or the threat classifications allocated. These differences, however, are not consistent. In some countries, national or subsidiary legislation may be more comprehensive, stringent or up to date than the IUCN Red Lists, but in other countries and jurisdictions the reverse applies, especially in developing nations. In this analysis, therefore, we relied on the IUCN Red List as the only standardised international database available.

Furthermore, our assessment made no attempt to evaluate the accuracy of the threatened species classification used but focused simply on where threatened species occurred in protected areas and the degree to which these areas were funded through tourism. Similar global assessments using these data sets have recently been completed for mammals and frogs [29], [30] and these data are widely used for other global threatened species assessments [1], [4], [10]. This is less of a limitation for birds than for mammal and frog species [29], [30], because in the case of birds, data from IUCN are supplemented by those from BirdLife International. For frogs [30], the shortcomings of subpopulation data forced the authors to rely on an alternative and less precise accounting metric, proportion of known range. For mammals [29], data on *S* and *R* were available jointly for only 90 of 1131 candidate species, which included vulnerable as well as critically endangered and endangered species. The calculations of *T* presented here for threatened bird species are thus the most comprehensive yet available for any major taxonomic group.

For the future conservation of threatened bird species, two key issues emerge from this first section of our analyses. (a) The limitations in available data demonstrate the importance of continuing research on bird species populations, distributions, ecology and threats. (b) The high proportions of threatened bird species (43%) which do not currently occur in any protected area worldwide demonstrates the importance of promoting biodiversity conservation across both public and private lands, even where expansion of the formal protected area network is not feasible. (c) Data on the joint distribution of critically endangered and endangered bird species, as highlighted here, could be used to direct conservation funding efficiently, thereby maximising overall threat reduction per unit expenditure.

Our second set of findings relates to the role of tourism revenue contributions to conservation of threatened bird species occurring at least in part within protected areas. Our results identify 41 species where at least 10% of each global population relies on tourism revenue. Naturally, these calculations are also subject to limitations in the scope, accuracy and reliability of financial data in the budgets of protected area agencies. Only a few countries produce and publish transparent, timely and externally audited annual financial statements for their protected area agencies that distinguish different revenue sources. Therefore, if anything, our findings underestimate the importance of tourism revenue for threatened birds in protected areas. When financial data become available for more protected area networks in more countries, a revised analysis would potentially identify a greater number of threatened species that are dependent on tourism revenues.

Despite these shortcomings in detailed data, our findings are broad and robust enough to demonstrate the important role of tourism in funding the conservation of threatened bird species. This reflects changing patterns in protected area funding more generally, over recent decades. Globally, government budget allocations are still the principal source of protected area management funds [13], [21]. However, many protected area agencies increasingly rely on tourism revenues to supplement or replace government allocations (Table 2) [19]. Tourism thus contributes to the conservation of threatened bird species, especially in developing nations with high biodiversity.

The other side of this coin, however, is that threatened bird species protected in parks are now subject to risks associated with the volatility of global tourism markets in addition to those already imposed by direct disturbance impacts [12], [28]. This is more of an issue for parks agencies in developing nations operating with less reliable government funding [13], [21], [37]–[40]. In addition, raising revenue from tourism to public protected areas may rely on charging user fees for visitors, and this is subject to equally volatile political and economic constraints [12], [13], [21]. In addition, for some countries, tourism revenues raised in protected areas not retained by the parks agencies at all. Such challenges are less problematic when tourism and conservation action occurs on private land, as has been demonstrated in southern Africa [16], [41]–[44]. For threatened bird species, the contributions of private enterprise have been quantified for only a few cases, notably the Seychelles White-eye on North Island and the Resplendent Quetzal (*Pharomachrus mocinno*) in the Monteverde Cloud Forest Reserve [6], [34].

The broad accounting approach taken here simply quantifies the degree to which tourism to protected areas contributes to funding conservation of threatened bird species. A number of more detailed or sophisticated approaches may also be feasible. 1. Differentiation by land tenure, distinguishing public, communal and private lands. 2. An accounting approach which calculates net contributions [45], allowing for negative environmental impacts [27], [28] as well as positive conservation measures, and distinguishing high-impact park-based mass tourism from low-impact park-based nature tourism. 3. A more detailed approach which relies on ecological modelling rather than purely on accounting. 4. A narrower focus, on the bird-watching or avitourism subsector specifically, as below.

Birdwatching is a significant and expanding subsector of the tourism industry. Avitourists travel either to see particular bird species, especially those that are rare or threatened; or to visit areas with high endemism and high diversity of bird species [46]–[52]. As shown here, many threatened bird species currently occur only outside protected areas, and this includes species attractive to birdwatchers. Specialist avitourism may thus create further incentives to expand the protected area network, or improve bird conservation on lands outside that network. In particular, this includes Important Bird Areas, designated in part due to the presence of threatened bird species. The role of avitourism in contributing to bird conservation through mechanisms such as Important Bird Areas merits further attention.

In conclusion, despite the dependence of tourism revenues on market conditions, and the risk associated with such dependence, we demonstrate that tourism revenue to protected areas makes a significant contribution to the conservation of threatened bird species, comparable to that for threatened mammals and frogs [29], [30]. While government funding remains a critical source of funding for many protected areas and conservation globally, we propose that this could be increased by promoting tourism on both public and private lands. Specialist niche tourism markets such as avitourism may provide further incentives to promote sustainable conservation tourism, particularly in those habitats identified as Important Bird Areas.

## Supporting Information

Table S1 **Proportional contributions of tourism to protected area budgets by country. All figures in local currencies at publication date of source.**
(DOCX)

Table S2 **Remaining global populations for critically endangered (CR) and endangered (EN) bird species surviving in only a single PA.**
(DOCX)

Table S3 **Distribution of critically endangered (CR) and endangered (EN) bird species by country.**
(DOC)
