# Supplementary material for: Tourism Revenue as a Conservation Tool for Threatened Birds in Protected Areas
Source: PLoS One. 2013 May 8;8(5):e62598. doi: 10.1371/journal.pone.0062598 (PMC3648576; doi:10.1371/journal.pone.0062598)
Supplement: Table S1 — Proportional contributions of tourism to protected area budgets by country. All figures in local currencies at publication date of source. (DOCX) [file pone.0062598.s001.docx]

Table S1. Proportional contributions of tourism to protected area budgets by country. All figures in local currencies at publication date of source.

| Country | Government PA Budget | Tourism Revenue | Donor/Other Funding | Total Budget | Proportion from Tourism | Source |
| --- | --- | --- | --- | --- | --- | --- |
| Argentina | 16,610,320 | 8,297,163 | 6,402,101 | 31,309,584 | 26.5 | Bovarnick *et al.* 2010 |
| Australia | 1,174,133,308 | 138,595,744 | 154,408,041 | 1,467,137,094 | 9.4 | Australian Conservation Agency reports (2006-2009) |
| Bolivia | 73,041 | 414,256 | 4,615,356 | 5,102,653 | 8.1 | Bovarnick *et al.* 2010 |
| Botswana | 1,066,808 | 7,444,288 | 671,742 | 9,182,837 | 81.1 | Financial reports - Botswana reserves |
| Brazil | 104,691,819 | 8,156,681 | 20,566,539 | 133,415,039 | 7.8 | Bovarnick *et al.* 2010 |
| Canada | 662,994,000 | 105,379,000 |  | 768,373,000 | 13.7 | Parks Canada Performance report 2010 |
| Chile | 5,705,515 | 3,488,824 |  | 9,194,339 | 37.9 | Bovarnick *et al.* 2010 |
| Colombia | 12,600,584 | 1,534,291 | 6,031,386 | 20,166,261 | 7.6 | Bovarnick *et al*. 2010 |
| Costa Rica | 14,302,092 | 5,398,612 | 9,945,245 | 29,645,949 | 18.2 | Bovarnick *et al*. 2010 |
| Cuba | 2,259,551 | 843,733 | 13,832,772 | 16,936,056 | 5.0 | Bovarnick *et al*. 2010 |
| Dominican Republic | 7,103,393 | 1,643,612 | 1,633,066 | 10,380,071 | 15.8 | Bovarnick *et al*. 2010 |
| Ecuador | 1,160,000 | 1,100,000 | 1,717,600 | 3,977,600 | 27.7 | Bovarnick *et al*. 2010 |
| El Salvador | 395,404 | 26,454 | 3,381,367 | 3,803,225 | 0.7 | Bovarnick *et al*. 2010 |
| Guatemala | 4,353,715 | 3,707,295 | 3,985,790 | 12,046,800 | 30.8 | Bovarnick *et al*. 2010 |
| Honduras | 677,057 | 1,032,265 | 2,413,230 | 4,122,552 | 25.0 | Bovarnick *et al*. 2010 |
| Kenya | 743,480,500 | 2,116,934,500 | 342,829,000 | 3,203,244,000 | 66.1 | KWS annual reports (2007/08) |
| Madagascar | 288,000 | 204,000 | 3,600,000 | 4,092,000 | 5.0 | Mansourian and Dudley 2008 |
| Mauritius* |  |  |  |  | 0 | Kevin Ruhomaun pers comm 2011 |
| Mexico | 49,046,698 | 4,740,532 | 26,427,009 | 80,214,239 | 5.9 | Bovarnick *et al*. 2010 |
| Namibia | 136,700,000 | 16,600,000 | 33,000,000 | 186,300,000 | 8.9 | Turpie *et al*. 2010 |
| Nepal | 213,228,510 | 117,898,991 |  | 331,127,501 | 35.6 | DNPWC annual report 2008 |
| New Zealand | 280,629,000 | 13,927,000 | 17,915,000 | 312,471,000 | 4.5 | DOC annual report 2009 |
| Nicaragua | 576,337 | 441,838 | 4,323,202 | 5,341,377 | 8.3 | Bovarnick *et al*. 2010 |
| Panama | 1,132,000 | 1,244,755 | 7,130,193 | 9,506,948 | 13.1 | Bovarnick *et al*. 2010 |
| Paraguay | 257,466 | 5,866 | 977,333 | 1,240,665 | 0.5 | Bovarnick *et al*. 2010 |
| Peru | 1,810,016 | 2,023,100 | 9,233,984 | 13,067,100 | 15.5 | Bovarnick *et al*. 2010 |
| Philippines | 45,415,544 | 51,269,283 |  | 96,684,827 | 53.0 | Carlo Custudio pers comm 2011 |
| Seychelles+ |  |  |  |  | 84.5 | Seychelles Island Foundation 2007; 2008 |
| South Africa | 662,483,828 | 724,398,339 | 146,484,530 | 1,533,366,696 | 47.2 | SANParks, ECPB, and Ezemvelo KZN Wildlife Annual reports |
| Tanzania | 105,775,369,000 | 61,200,000,000 |  | 166,975,369,000 | 36.7 | TANAPA annual reports |
| Thailand | 35,486,343 | 11,863,163 | 841,136 | 48,190,642 | 24.6 | GEF PA development report 2009 |
| United States | 3,160,000,000 | 251,200,000 |  | 3,411,200,000 | 7.4 | J. A. Pendry pers comm 2011 |
| Uruguay | 606,000 | 66,000 | 144,000 | 816,000 | 8.1 | Bovarnick *et al*. 2010 |
| Venezuela | 20,628,837 | 2,567,260 | 0 | 23,196,097 | 11.1 | Bovarnick *et al*. 2010 |
| Zambia | 7,207 | 25,742 | 20,347 | 53,295 | 48.3 | ZAWA annual report 2007 |

* Black River Gorges NP only. +Aldabra Atoll only.
