# Supplementary material for: Tourism Revenue as a Conservation Tool for Threatened Birds in Protected Areas
Source: PLoS One. 2013 May 8;8(5):e62598. doi: 10.1371/journal.pone.0062598 (PMC3648576; doi:10.1371/journal.pone.0062598)
Supplement: Table S2 — Remaining global populations for critically endangered (CR) and endangered (EN) bird species surviving in only a single PA. (DOCX) [file pone.0062598.s002.docx]

Table S2. Remaining global populations for critically endangered (CR) and endangered (EN) bird species surviving in only a single PA.

| Species Name | Common Name | IUCN | Protected Area | Country | Population |
| --- | --- | --- | --- | --- | --- |
| *Aceros narcondami* | Narcondam Hornbill | EN | Narcondam Island Wildlife Sanctuary | India | 320-340 |
| *Aratinga brevipes* | Socorro Parakeet | EN | Revillagigedo Islands Biosphere Reserve | Mexico | 300 |
| *Buteo ridgwayi* | Ridgway's Hawk | CR | Los Haitises National Park | Dominican Republic | 160-240 |
| *Camarhynchus pauper* | Medium Tree-finch | CR | Galapagos National Park | Ecuador | 1660 |
| *Cyanoramphus cookii* | Norfolk Island Parakeet | EN | Norfolk Island National Park | Australia | 200-300 |
| *Diomedea amsterdamensis* | Amsterdam Albatross | CR | French Southern and Antarctic Lands | France | 130 |
| *Hylonympha macrocerca* | Scissor-tailed Hummingbird | EN | Paria Peninsula National Park | Venezuela | 10000-19999 |
| *Laterallus tuerosi* | Junin Rail | EN | Junin National Reserve | Peru | 1000-2499 |
| *Malaconotus alius* | Uluguru Bush-shrike | CR | Ulguru Forest Reserve | Tanzania | 2400 |
| *Mimus graysoni* | Socorro Mockingbird | CR | Revillagigedo Islands Biosphere Reserve | Mexico | 290-420 |
| *Mimus melanotis* | San Cristobal Mockingbird | EN | Galapagos National Park | Ecuador | 8000 |
| *Mimus trifasciatus* | Floreana Mockingbird | CR | Galapagos National Park | Ecuador | 100 |
| *Myadestes palmeri* | Puaiohi | CR | Alakai Wilderness Preserve | United States (Hawaii) | 200-500 |
| *Myiotheretes pernix* | Santa Marta Bush-tyrant | EN | Sierra Nevada de Santa Marta Biosphere Reserve | Colombia | 1000-2499 |
| *Oreomystis bairdi* | Akikiki | CR | Alakai Wilderness Preserve | United States (Hawaii) | 780-1840 |
| *Papasula abbotti* | Abott’s Booby | EN | Christmas Island National Park | Australia | >6000 |
| *Phoebastria irrorata* | Waved Albatross | CR | Galapagos National Park | Ecuador | 34700 |
| *Podiceps taczanowskii* | Junin Grebe | CR | Junin National Reserve | Peru | 217-304 |
| *Polioptila clementsi* | Iquitos Gnatcatcher | CR | Reserva Nacional Allpahuayo-Mishana | Peru | 50-249 |
| *Pterodroma axillaris* | Chatham Petrel | EN | South East Island (Rangatira) Nature Reserve | New Zealand | 1000-1100 |
| *Pterodroma madeira* | Zino's Petrel | EN | Parque Natural da Madeira | Portugal | 130-160 |
| *Rowettia goughensis* | Gough Bunting | CR | Gough Island Nature Reserve | Saint Helena, Ascension and Tristan da Cunha | 1000 |
| *Sephanoides fernandensis* | Juan Fernandez Firecrown | CR | Juan Fernandez National Park | Chile | 2500-3000 |
| *Telespiza ultima* | Nihoa Finch | CR | Hawaiian Islands National Wildlife Refuge | United States (Hawaii) | 2060-3550 |
| *Todiramphus gambieri* | Tuamotu Kingfisher | CR | Fakarava Biosphere Reserve | French Polynesia | 125 |
| *Zosterops albogularis* | White-chested White-eye | CR | Norfolk Island National Park | Australia | <50 |
| *Zosterops rotensis* | Rota Bridled White-eye | CR | Sabana Protected Area  (Community Managed) | Northern Mariana Islands | 1100 |
| *Zosterops tenuirostris* | Slender-billed White-eye | EN | Norfolk Island National Park | Australia | 2000 |
