# Supplementary material for: Tourism Revenue as a Conservation Tool for Threatened Birds in Protected Areas
Source: PLoS One. 2013 May 8;8(5):e62598. doi: 10.1371/journal.pone.0062598 (PMC3648576; doi:10.1371/journal.pone.0062598)
Supplement: Table S3 — Distribution of critically endangered (CR) and endangered (EN) bird species by country. (DOC) [file pone.0062598.s003.doc]

Table S3. Distribution of critically endangered (CR) and endangered (EN) bird species by country.

| Country | Number of protected areas with CR and/or EN birds | Number of CR bird species in protected areas | Number of EN bird species in protected areas | Number of CR and EN species found in only one protected area | Number of protected areas with more than one CR or EN bird species |
| --- | --- | --- | --- | --- | --- |
| Algeria | 1 |  | 1 |  |  |
| Angola | 1 |  | 1 |  |  |
| Argentina | 6 | 1 | 2 |  |  |
| Australia | 12 | 2 | 10 | 4 | 2 |
| Bangladesh | 2 |  | 1 |  |  |
| Bermuda | 1 |  | 1 |  |  |
| Bolivia | 10 | 1 | 9 |  | 3 |
| Brazil | 65 | 12 | 20 |  | 14 |
| Burundi | 1 |  | 1 |  |  |
| Cambodia | 10 | 1 | 2 |  | 4 |
| Cameroon | 3 |  | 3 |  | 1 |
| Canada | 1 |  | 1 |  |  |
| Chile | 2 | 2 | 1 | 1 | 1 |
| China | 19 |  | 9 |  | 3 |
| Colombia | 43 | 7 | 24 | 1 | 14 |
| Cook Islands | 1 |  | 1 |  |  |
| Costa Rica | 4 |  | 3 |  | 2 |
| Côte d'Ivoire | 6 |  | 2 |  | 1 |
| Cuba | 4 | 1 | 4 |  | 1 |
| Democratic Republic of Congo | 2 |  | 2 |  |  |
| Dominica | 2 |  | 1 |  |  |
| Dominican Republic | 3 | 1 | 3 | 1 | 2 |
| Ecuador | 22 | 7 | 14 | 4 | 4 |
| Ethiopia | 2 |  | 2 |  |  |
| Fiji | 3 |  | 1 |  |  |
| France | 3 | 2 | 4 | 1 | 1 |
| French Polynesia | 2 | 1 | 1 | 1 |  |
| Guatemala | 2 |  | 2 |  | 1 |
| Haiti | 1 |  | 1 |  |  |
| Honduras | 5 | 1 | 2 |  |  |
| India | 12 | 2 | 4 | 1 | 1 |
| Indonesia | 35 | 5 | 15 |  | 6 |
| Jamaica | 1 |  | 1 |  |  |
| Japan | 7 | 1 | 3 |  |  |
| Kenya | 1 |  | 1 |  |  |
| Laos | 1 |  | 1 |  |  |
| Madagascar | 17 | 1 | 5 |  | 3 |
| Mauritius | 1 | 1 | 3 |  | 1 |
| Mexico | 9 | 2 | 8 | 2 | 3 |
| Morocco | 1 | 1 |  |  |  |
| Myanmar | 3 | 1 | 2 |  | 1 |
| Nepal | 1 |  | 1 |  |  |
| New Caledonia | 3 | 2 | 1 |  |  |
| New Zealand | 29 | 4 | 14 | 1 | 8 |
| Nicaragua | 1 |  | 1 |  |  |
| Nigeria | 1 |  | 1 |  |  |
| North Korea | 5 |  | 2 |  |  |
| Northern Marianas | 1 | 1 |  | 1 |  |
| Palau | 1 |  | 1 |  |  |
| Panama | 6 |  | 2 |  |  |
| Paraguay | 3 |  | 2 |  |  |
| Peru | 15 | 3 | 16 | 3 | 6 |
| Philippines | 13 | 4 | 7 |  | 4 |
| Pitcairn Islands | 1 |  | 1 |  |  |
| Portugal | 2 |  | 2 | 1 |  |
| Puerto Rico | 3 | 2 |  |  |  |
| Russia | 11 |  | 4 |  | 2 |
| Rwanda | 1 |  | 1 |  | 1 |
| Saint Helena, Ascension and Tristan da Cunha | 2 | 2 | 4 | 1 | 2 |
| Saint Lucia | 3 |  | 1 |  |  |
| Samoa | 1 |  | 1 |  |  |
| Seychelles | 9 | 1 | 5 |  | 3 |
| Sierrra Leone | 2 |  | 2 |  |  |
| Solomon Islands | 1 |  | 1 |  |  |
| Somalia | 1 |  | 1 |  |  |
| South Africa | 7 |  | 3 |  | 1 |
| Spain | 3 |  | 2 |  |  |
| Sri Lanka | 5 |  | 2 |  |  |
| Suriname | 1 |  | 1 |  |  |
| Tanzania | 10 | 2 | 5 | 1 | 2 |
| Thailand | 3 |  | 3 |  |  |
| Timor Leste | 1 | 1 |  |  |  |
| Uganda | 6 |  | 3 |  |  |
| United States | 24 | 6 | 9 | 3 | 6 |
| Uruguay | 1 |  | 1 |  |  |
| Venezuela | 10 |  | 10 | 1 | 2 |
| Vietnam | 5 |  | 5 |  | 1 |
